# Work-Family Conflict, Enrichment, and Adolescent Academic Adjustment in Dual-Earner Family

**DOI:** 10.3389/fpsyg.2021.712954

**Published:** 2021-12-03

**Authors:** Xiaoli Wang, Lijin Zhang, Xiujuan Wu, Min Zhao

**Affiliations:** ^1^School of Psychology, Shaanxi Normal University, Xi’an, China; ^2^School of Elementary Education, Changji University, Changji, China; ^3^Shaanxi Provincial Key Research Center of Child Mental and Behavioral Health, Xi’an, China; ^4^Shaanxi Key Laboratory of Behavior and Cognitive Psychology, Xi’an, China

**Keywords:** work-family conflict, work-family enrichment, parental educational expectation, academic adjustment, perceived educational expectations

## Abstract

There is ample evidence that work-family conflict (WFC) and work-family enrichment (WFE), respectively, have detrimental and beneficial impacts on the functioning of couples, families, and children. In this study, cross-sectional data from 2,136 dual-earner families in China, including parents and their children (51.2% girls, ages: 11.6–19.3 years), were used together with Actor-Partner Interdependence Model-Structural Equation Modeling (APIM-SEM) to test the hypothesis that work-family spillover can impact academic adjustment in adolescents through parental educational expectations and perceived educational expectations. The results of this analysis suggested that academic adjustment among adolescents is primarily influenced by maternal work-family experiences, such that maternal but not paternal WFC can impact academic adjustment in adolescents through parental educational expectations and perceived educational expectations. Maternal WFE was found to be indirectly associated with the academic adjustment in adolescents as a result of actual and perceived educational expectations. Additionally, we observed a significant effect of maternal WFC on the educational expectations of fathers within couple-relationship dyads. These results underscore the importance of the work-family interface as a factor that shapes the overall family health and associated outcomes, especially the importance of maternal work-family experiences in this context. Interventions that aim to promote more positive maternal work environments are thus likely to yield greater benefits for their children and families. Overall, these data indicate that work-family spillover is a core determinant of adolescent development, which warrants further study.

## Introduction

Early adolescence corresponds to the transition from childhood to adulthood, with concomitant shifts in academic achievement, motivation, and engagement (Shim and Finch, 2014). These transitions are often challenging for adolescents, and experiences during this period can profoundly impact their academic and emotional adjustment (Duineveld et al., 2017). Adolescents are at risk of dropping out of school and/or becoming maladjusted to this learning environment (Anderman, 2003).

At a basic level, academic adjustment refers to the degree to which students manage different social, psychological, and academic challenges at school. Such an adjustment can be impacted by both intrinsic social or psychological factors and by extrinsic factors such as work-family circumstances and the physical and mental state of the parent of a given student (Fuligni et al., 2001; Tseng, 2004; Brière et al., 2015; Arjanggi and Kusumaningsih, 2016). Higher levels of work-family conflict (WFC) have been reported to be associated with increased academic pressure and a higher risk of burnout (Engels et al., 2019). The negative impacts of such work-family spillover can also indirectly affect such academic pressure through effects on parental educational expectations and associated changes in parental mental state (Berchiatti et al., 2020; Ebeling, 2020).

The reasons why some adolescents can excel in academic settings whereas others experience significant difficulties in the same setting are not fully understood. However, adolescent self-perceptions, including perceptions regarding parental beliefs and behaviors, are known to be key predictors of academic success. Thus, the differences in individual perceptions may ultimately shape the ability of students to effectively adapt to their academic environment (Dotterer and Lowe, 2015; Duineveld et al., 2017).

While prior studies on work-family interactions have demonstrated that WFC can negatively impact adolescent developmental outcomes through parenting practices and experiences, there have been far fewer studies exploring the potential benefits of work-family enrichment (WFE) on academic cognition and associated adolescent behaviors. By better understanding these interactions, it may be possible to design novel interventions capable of aiding adolescent academic adjustment. Thus, this study was designed to explore the association between parental work-family spillover and academic adjustment and to establish whether this relationship was mediated by parental educational expectations and/or perceived parental educational expectations.

### Work-Family Spillover and Academic Adjustment: Differences in Parental Roles

Work-family spillover refers to a measurement of the interplay between work and family roles that examines the extent to which these roles are enriching or conflicting (Shaffera et al., 2001; Greenhaus and Foley, 2007). In this contextual framework, WFC is considered to be a process whereby demands in a given domain deplete personal resources and interfere with accomplishments in other domains of life, while WFE refers to a process of resource accumulation wherein work-derived resources ultimately improve experiences associated with a distinct social role (Greenhaus and Beutell, 1985; Powell and Greenhaus, 2006; ten Brummelhuis and Bakker, 2012).

Such work-family spillover can impact many aspects of childhood development and adolescent adjustment (Bronfenbrenner, 1986). For example, conflicts between parental responsibilities in the workplace and familial contexts have been shown to negatively impact adolescent adaptive outcomes. Such conflicts can, for example, lead to increased academic pressure and/or reduced academic performance collectively referred to as academic maladjustment (Greenfield and Yan, 2006; Fosco and Grych, 2008; Rönkä et al., 2018; Vahedi et al., 2019). WFC has been linked to poorer academic performance, worse learning outcomes, and decreased school achievement (Shim and Finch, 2014; Yang et al., 2015; Graber et al., 2016). However, further research regarding the potential positive impacts of work-family spillover remains to be conducted. Indeed, there is some evidence that work-derived rewards and enrichment can improve parenting quality (Eby et al., 2005; Cho and Allen, 2012). For example, parents with higher salaries are more likely to create an intellectually stimulating environment for their children in order to support their education (McNall et al., 2010). Such support has been referred to as a “hidden curriculum” that contributes to overall improvements in academic adjustment (Mekonnen, 2017). A better understanding of the negative and positive effects of such a spillover is thus critical to the shaping of educational practices aimed at optimizing adolescent development.

In present China, both mothers and fathers must often balance work and childcare obligations. In dual-earner families, however, gender roles are likely to be more traditional such that fathers serve as the primary financial providers while mothers are mainly responsible for the children (Fan and Chen, 2020; Xia et al., 2020). These gender-related differences may lead parents to experience work-family spillover in different ways, given their distinct perceptions regarding work and family, although few studies to date have explored this possibility. The maternal workforce participation is not linked to a concomitant reduction in housework or childcare obligations as is the case for fathers, potentially reducing marital and work satisfaction and ultimately increasing maternal WFC (Crouter and Bumpus, 2001; Nilsen et al., 2016; Kuo et al., 2018). Mothers are also more likely to play a direct role in the daily management of family life such that maternal work-family experiences may have a greater impact on family function and child developmental outcomes (Cooklin et al., 2015).

While there is a work-family theoretical basis (Demerouti et al., 2005) to expect fathers in order to experience less distress than mothers, they may nonetheless experience greater WFC owing to desires of having a fulfilling career and a quality family life simultaneously (Shockley et al., 2017; Kuo et al., 2018). Paternal WFC has previously been shown to be damaging to early childhood development, with resultant outcomes persisting into adolescence (Bronte-Tinkew et al., 2006; Lee et al., 2012). Spousal work-family experiences can also predict individual experiences (Day and Chamberlain, 2006) and views regarding their children (Matias and Recharte, 2020), with such effects being most pronounced for fathers, who may be more prone to experience stronger partner crossover influences as their parenting role is less well scripted than the maternal role such that they are more susceptible to contextual influences (Belsky et al., 1991).

### Mediating Impact of Educational Expectations

Parental educational beliefs and practices can play a key role in influencing adolescent academic progress (Beal and Crockett, 2010; Chen et al., 2020; Fang et al., 2020), with parental educational expectations playing a particularly profound role in shaping social and academic adjustment in adolescent children (Phillipson and Phillipson, 2007; August et al., 2012). Parents with higher educational expectations are more likely to be supportive of the education of their children (Hinnant et al., 2009; Chen et al., 2020), leading to higher average scores from standardized tests, better average grades, and lower rates of school dropout relative to the children of parents with low educational expectations (Davis-Kean, 2005; Ravindran and Kalpana, 2012). These results suggest that parental educational expectations can serve as a robust family-level predictor of academic achievement among adolescents (Lazarides et al., 2016; Kang et al., 2020).

In general, parents typically believe that better educational achievement is directly linked to better career success and social status, and as such, a rising fraction of parents expect their children to receive a better education and to thereby attain a better career (Chen et al., 2020; Chan and Li, 2020; Hawkley et al., 2020; Li, 2020).

The cognitive attitudes and abilities of adolescents differ from those of their parents, as does their understanding of the associated environment (Lazarides et al., 2016). As such, they actively construct distinct views of themselves and the future based upon information derived from their parents. Adolescent perceptions of parental educational expectations are thus likely to be distinct from the actual expectations of their parents (Kuperminc et al., 2008; Beal and Crockett, 2010).

Prior empirical studies have demonstrated a positive correlation between parental expectations and educational beliefs and attitudes of adolescents. However, if adolescents perceive that the educational expectations of their parents exceed their personal expectations, maladjustment may occur (Benner and Wang, 2014; Wang and Benner, 2014). Adolescent perceptions of parental educational expectations can thus be considered a form of reactive evaluation in accordance with identity control theory (Burke, 1996). According to this theory, when there is a misalignment between these evaluations, individuals can seek to remediate these inconsistencies *via* output behaviors, with maladjustment occurring when these behaviors fail to achieve the desired outcome (Eccles and Wigfield, 2002). As such, the extent to which the perceived educational expectation maybe was a key factor to the academic adjustment of the adolescents.

Prior work-family studies suggested that parental education level, parental occupation, family assets, and work-family spillover can all impact educational attitudes (Porfeli et al., 2008; Fang et al., 2020). WFC can also negatively impact parental educational attitudes, and parental educational expectations serve as a key metric for these attitudes (Graves et al., 2007). A lack of time or energy attributable to work-life spillover may impact parental mental status such that it indirectly impacts academic adjustment (Bilodeau et al., 2020).

While parental expectations represent a mental state that cannot directly affect adolescent cognition, behavior, or performance, they can mediate the relationship between work-family spillover, academic pressure, academic burnout, and academic self-efficacy (Hinnant et al., 2009; Beal and Crockett, 2010; de Haan et al., 2013; Jiang and Dong, 2020). However, relatively little is known regarding the manner whereby work-family experiences impact adolescent adjustment, particularly when simultaneously considering parental education expectations and perceived educational expectations. Therefore, we assessed whether educational expectations function as an important mechanism explaining the impact of work-family spillover on adolescent academic adjustment.

### The Current Study

Most studies of relationships between adolescents and their parents have focused on single-informant designs that utilize adolescent self-reported information pertaining to parental behaviors, with less distinction being made between maternal and paternal behaviors. Given that work-family spillover for mothers and fathers can have significant but distinct impacts on academic adjustment and development, there is thus a major gap in the associated literature. The primary goal of this study was, thus, to address these gaps by investigating relationships between parental reports regarding work-family spillover, educational expectations (both maternal and paternal), and adolescent reports regarding academic adjustment. It is important that both maternal and paternal factors should be considered at the same time in order to ensure that important factors influencing academic performance are not overlooked.

In this study, we employed an Actor-Partner Interdependence Model-Structural Equation Modeling (APIM-SEM) approach to test model to study these relationships in greater detail such that maternal and paternal results can be compared effectively. We hypothesized that parental WFC and WFE would be significantly associated with adolescent academic adjustment. Using our model, we sought to test whether these associations were indirectly mediated by parental educational expectations and adolescent perceptions thereof. We also assessed whether these associations differed as a function of parental role and examined whether there were actor-partner interdependent associations of spouse educational expectations.

Finally, the mothers as caregivers is highly pervasive, giving the flow-on implications the role of mothers has to families, family relationships, parent-child interactions, and, ultimately, in the development of children (Matias and Recharte, 2020). Thus, we expected that the hypothesized paths would be stronger for mothers than fathers at any time.

## Materials and Methods

### Participants and Procedure

This study was approved by the University Research Ethics Committee of Shaanxi Normal University (Xi’an, China). The current research was conducted at five full schools in Jiangxi Province, China. Ten classes from grades 5–9 were randomly selected from each school to participate in this study. In this research, both adolescents and their parents were invited to participate. They were told that their participation was voluntary and that they could choose not to participate if they desired. The anonymity of participants was guaranteed, and they were assured that information would be used only for research purposes.

Parents provided written informed consent for their participation and the participation of their children. The students were tested by taking one class as a unit to evaluate the academic adjustment of adolescents during a single 45-min classroom session. Adolescents brought the questionnaires to their parents so that they could complete the questionnaires at home. Parents were asked to complete questionnaires at home and return the sealed questionnaires within 1 week of receipt to their headteachers. Mothers and fathers were asked to complete questionnaires on WFC, WFE, demographic characteristics, and educational expectations separately from each other. Families with more than one child in the target age range were instructed to choose only one of the tested children to fill out the questionnaire. The participated adolescents and their parents were awarded 50 ¥, respectively.

A total of 2,680 students were approached: 269 students were excluded because one or both parents were unemployed, 124 students were excluded because they did not complete the questionnaires, and 151 students were excluded because they were non-cohabiting with a mother and father figure for at least 2 years. The demographic characteristics of adolescents, including age, gender, and siblings, were collected using a self-reported questionnaire. Adolescents were 11.6–19.3 years of age: 14.1% were fifth graders, 17.7% were sixth graders, 28.9% were seventh graders, 22.3% were eighth graders, and 17.0% were ninth graders; 51.2% were girls; *M* = 13.11, *SD* = 1.28. In total, we approached 2,680 parents: 388 parents were excluded because they did not complete the questionnaires and 156 parents were excluded because of lacking of data on one parent. The final sample consisted of 2,136 families, including 2,136 adolescents, 2,136 fathers, and 2,136 mothers.

Families varied in their number of children: 51% of the families had two children, 12% of the families had three children, 31% of the families had only one child, and 6% of the families had 4 children or more. For these participants, the majority of mothers and fathers reported being biological parents of the participating adolescents (>91%).

### Measures

#### Demographic Characteristics

A self-reported questionnaire was used to collect the educational background of parents, age (mean age of mothers = 40.54 years, *SD* = 3.48; mean age of fathers = 41.92 years, SD = 2.93), and working hours. Regarding the demographic characteristics of parents, 27.7% of fathers and 25.5% of mothers had a high school education or higher and 51.8% of fathers and 48.4% of mothers had a secondary education diploma: 13.1% of families reported income < ¥ 5,000 per month, 34.2% between ¥ 5,000 and ¥ 9,999 per month, 38.1% between ¥10,000 and ¥14,999 per month, and 14.6% > ¥ 15,000 per month. Regarding the working hours and overtime of fathers, 43.5% reported working 8 h/day and 32.7% working over 8 h/day. For mothers, 55.6% reported working 8 h/day and 26.9% working over 8 h/day. Notably, 13.5% fathers and 3.4% mothers reported working some hours at night. Almost one-third of parents reported working overtime and some fathers had to work late in the night.

#### Work-Family Conflict and Work-Family Enrichment

Parents reported WFC and WFE using the Work-Family Spillover Scale (Wayne et al., 2004). This questionnaire consisted of 16 items, describing two aspects from WFC (8 items, e.g., “My job reduces the effort I can give to activities at home”) and WFE (8 items, e.g., “The things I do at work help you deal with personal and practical issues at home”). The Chinese version of this questionnaire has been validated (Ma et al., 2018).

Parents indicated how often they had experienced each during the last month on a five-point Likert scale ranging from 1 (*all the time*) to 5 (*never*). Items were scored such that higher scores meant more conflict or enrichment. The Cronbach’s alpha coefficients of this study are 0.79 and 0.84 for both mothers and fathers, respectively.

#### Educational Expectations

Educational expectations were measured by the response of the father and mothers to the questions, “How far in school do you want your child to get?” and adolescent perceptions of educational expectations of their parents (“How far in school do you think your parent want you to get?”), answered on a Likert scale ranging from 1 (*less than high school*) to 6 (*more than postgraduate*) (Wang and Benner, 2014).

#### Academic Adjustment

This study used the 24-item Adaptive Learning Scales (Midgley et al., 2000) to assess the academic adjustment of adolescents. This scale included three subscales: academic pressure (e.g., “Having to study things you do not understand”), academic efficiency (e.g., “Well behaved in school”), and academic burnout (e.g., “My study is so poor that I really want to give it up”). For the above statements, responses ranged from 1 (*never true*) to 5 (*always true*), with items reverse-scored when necessary, and higher scores representing higher maladaptive. The Cronbach’s alpha coefficients of each subscale ranged from 0.70 to 0.83 in this study.

### Analytic Strategy

We first performed descriptive statistics and bivariate correlations between all variables using SPSS 25.0 (SPSS, 2014).

In the following step, we examined three models: (1) the mediating role of educational expectations and perceived parental educational expectation between the WFC of parents and academic adjustment of adolescents (Model 1), (2) the mediating role of parental educational expectations and perceived parental educational expectations between the WFE of parents and academic adjustment of adolescent (Model 2), and (3) the model combining WFC and WFE (Model 3). We also estimated the covariance between the educational expectations of fathers and mothers in all models in order to assure that the result is robust. AMOS 20.0 was used to account for the complex survey design of the data (Arbuckle, 2011).

Finally, to examine whether parental educational expectations and perceived educational expectations of adolescents could mediate the associations between WFC and WFE of parents and academic adjustment of adolescents, we used the Actor-Partner Interdependence Model-Structural Equation Modeling (APIM-SEM) model, which can be used to measure effects between two variables within an individual (i.e., actor effect) and simultaneously accounting for interpersonal relationship regarding the outcome of his/her partner (i.e., partner effect; Cook and Kenny, 2005). Therefore, the APIM-SEM model allows us to better understand the unique contributions of WFC and WFE the parents to their own (actor effect) and educational expectations of each other (partner effect), as well as the effects of these contributions on academic adjustment in adolescents.

We controlled for time-invariant characteristics, including age (in years), gender (1 = female and 0 = male), school (1 = primary school and 2 = secondary school), and socioeconomic status (SES). Parental educational attainment (high school education or higher and secondary education diploma, Chen et al., 2020) and monthly household income of 5,000 ¥ are indicators of SES.

## Results

### Descriptive Statistics and Correlations Among Study Variables

Table 1 illustrates the descriptive statistics and Pearson’s bivariate correlations for our variables. Consistent with our expectations, the academic adjustment of adolescents was negatively correlated with the WFC of parents and positively correlated with the WFE of parents. Parental educational expectations were positively correlated with their WFE and negatively correlated with their WFC. In addition, the perceived educational expectations of adolescents exhibited significantly positive correlations with educational expectations of their parents and significantly negative correlations with their academic adjustment.

**TABLE 1:** Means, standard deviations, and correlations among the study variables (*N* = 2136).

	***M* (*SD*)**	**1**	**2**	**3**	**4**	**5**	**6**	**7**	**8**	**9**	**10**
1. Grade	–	–									
2. Gender	–	−**0.01**	–								
3. SES	-0.03 (2.92)	−0.13^**^	**−−0.07** ^**^	–							
4. WFC (R-F)	2.51 (0.79)	0.04	–0.02	0.00	–						
5. WFC (R-M)	3.27 (0.83)	**0.10** ^**^	–0.01	−0.05^*^	**0.33** ^**^	–					
6. WFE (R-F)	2.51 (0.80)	0.02	–0.03	0.02	–0.03	–0.01	–				
7. WFE (R-M)	3.30 (0.83)	0.03	–0.02	0.00	−0.05^*^	0.03	**0.60** ^**^	–			
8. Educational expectation (R-F)	4.27 (0.98)	–0.02	0.00	**0.11** ^**^	−0.19^**^	−0.16^**^	**0.08** ^**^	**0.06** ^**^	–		
9. Educational expectation (R-M)	4.31 (0.97)	0.03	–0.01	**0.14** ^**^	−0.05^*^	**−−0.16** ^**^	**0.11** ^**^	**0.15** ^**^	**0.50** ^**^	–	
10. Perceived educational expectation	4.31 (1.16)	0.00	0.02	0.02	–0.04	–0.03	0.04	0.06^**^	0.16^**^	**0.31** ^**^	–
11. Academic adjustment	2.61 (0.43)	**0.18** ^**^	0.01	–0.03	**0.09** ^**^	**0.13** ^**^	−0.09^**^	−0.05^*^	−0.04^**^	–0.02	0.07^**^

WFC, work-family conflict; WFE, work-family enrichment; R-M, report by mother; R-F, report by father.*p < 0.05, **p < 0.01.

### Work-Family Conflict and Work-Family Enrichment of Parents and Academic Adjustment of Adolescents: The Mediating Role of Educational Expectations

Mediation analyses were conducted to examine the hypotheses that the work-family spillover of parents had implications for academic adjustment. The APIM-SEM model was used to assess the mediating effects of parental educational expectation and perceived educational expectation on this indirect path.

To examine whether parental educational expectations and perceived educational expectations of adolescents could mediate the associations between parental WFC and WFE and academic adjustment of adolescents, we estimated and compared three models: WFC (Model 1), WFE (Model 2), and combining Model 1 and Model 2 (Model 3). As shown in Table 2, Model 3 has better model fitting than Model 1 and Model 2. Therefore, we selected Model 3 as our final mediation model.

**TABLE 2:** Fit indices of the model of WFC, WFE, and comprehensive (*N* = 2,136).

**Model**	**χ^2^/*df***	**TLI**	**CFI**	**RMSEA**
Model 1 (WFC)	10.03	0.91	0.96	0.06
Model 2 (WFE)	5.39	0.97	0.98	0.05
Model 3 (Combined model)	3.04	0.92	0.97	0.04

Then, we tested the relationships between WFC of parents and educational expectation, and the result shows that the model with distinguishable members differed from that with indistinguishable members (Δχ^2^ = 20.53, *p* < 0.001). The results of the model comparison show that distinguishability existed between the educational expectations of mothers and fathers; i.e., the APIM-SEM model is supported for model testing, in which model parsimony can be considered for increasing model power.

**FIGURE 1:**
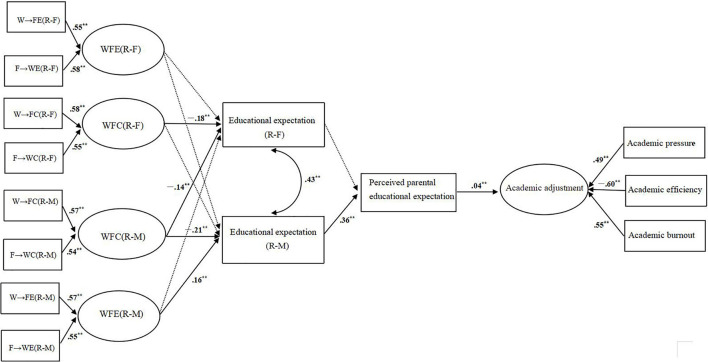
The mediated impact of parental educational expectation and perceived educational expectation on WFC, WFE, and academic adjustment, final model. W→FC, work→family conflict; F→WC, family→work conflict; W→FE, work→family enrichment; F→WE, family→work enrichment. Solid arrows indicate significant paths; dotted arrows indicate not significant paths: Standardized estimates are depicted. *p < 0.05, **p < 0.01.

As shown in Figure 1, WFC demonstrated significant actor effects on educational expectations in both mothers and fathers. The standardized actor effect was for father (β = −0.18, SE = 0.03, *p* < 0.001, 95% CI [−0.21, 0.09]) and for mother (β = −0.21, SE = 0.03, *p* = 0.001, 95% CI [−0.22, −0.11]), and WFC of mothers was negatively predicted with educational expectation of fathers (β = −0.14, SE = 0.03, *p* < 0.001, 95% CI [−0.17, −0.06]); however, we did not find significant effects of WFC of fathers on the educational expectation of mothers within the couple-relationship dyads. Meanwhile, only WFE of mothers displayed a significant positive effect on the educational expectations they hold (β = 0.16, SE = 0.03, *p* < 0.001, 95% CI [0.06, 0.19]). In other words, their educational expectation does not appear dependent on one another (see Figure 1).

This finding proved the possibility that parental roles may have different effects on the academic adjustment of adolescents. Then, we added adolescent’s perceived educational expectation of adolescents and in the next step of the models to test the extent to which associations between work-family spillover of parents and academic adjustment of the adolescent were mediated or independent.

Furthermore, our model examined the relations between WFC, the parental educational expectation of parents, perceived educational expectation of adolescents, and academic adjustment. For fathers, indirect effects were not significant. However, indirect effects were significant for the relations between WFC of mothers and academic adjustment of adolescents, *via* both educational expectation of mother and perceived educational expectation of adolescents (β = −0.02, 95% CI [−0.02, −0.01], *p* < 0.001) (for actor effect). Notably, WFC of mothers had a direct effect on the educational expectation of fathers (β = −0.01, 95% CI [−0.001, −0.002], *p* < 0.05) (for partner effect); however, the path coefficient of the relationship between paternal educational expectation and perceived educational expectation was not significant (β = −0.03, *p* = 0.54). In other words, the total effect of maternal WFC on academic adjustment *via* paternal educational expectation and perceived educational expectation of adolescents was non-significant.

Additionally, the indirect relations between WFE of fathers and academic adjustment are also not significant, and the indirect relations between WFE of mothers and academic adjustment of adolescents (β = 0.01, 95% CI [0.002, 0.001], *p* < 0.001) (for actor effect), *via* only educational expectation of mothers and perceived educational expectation of adolescents, were significant (see Figure 1).

## Discussion

First, this study is one of only a few, to our knowledge, to have examined the impacts of WFC and WFE on the outcomes of adolescents in China. In fact, according to the resources model (Hobfoll, 1989), it is necessary to consider both WFC and WFE in the study because the two processes often occur simultaneously (Vahedi et al., 2018; Matias and Recharte, 2020). This study contributes to the body of research by considering how the labor market plays a role on adolescent health *via* influences on the work and family functioning of parents. Second, the spillover-crossover model combines the spillover and crossover literature and theorizes that the experiences of job demands of individuals and resources will first spill over to their family domain and then cross over to their spouses and children (Shimazu et al., 2011). Our findings indicate that such work-family spillover of parents has an impact on adolescent adjustment. Finally, these results also underscore a strong correlation between parental work-family spillover and academic adjustment in adolescents, while supporting the mediating roles whereby parental educational expectations and perceived educational expectations of adolescents impact this relationship. The findings replicate and expand previous studies on the role of work-family resources of parents as main correlates of youth development. Moreover, regarding interactions between the work-family interface and adolescents, mothers seem to play a more important role than fathers (Greenhaus and Powell, 2006; Matias and Recharte, 2020) and interventions for mothers might be more effective.

### Work-Family Conflict and Academic Adjustment

The results of the model in this study suggested that parental WFC negatively affected the educational expectations they hold, and maternal WFC does not only affect the mother but also her spouse, however (Greenhaus and Beutell, 1985; Coplan and Weeks, 2009). If parents are depleted of resources in the workplace, they will find it more difficult to focus on the child or be mentally available during parent-child interactions. As such, WFC likely will impair the availability of the caregivers to the child, such as low expectations and rough parenting. Similarly, mothers who feel overwhelmed by forms of WFC such as overtime or childcare responsibilities may place increased demands on fathers, thereby influencing their expectations.

In addition, we also found an indirect impact of maternal WFC but not paternal WFC on academic adjustment in adolescents (Borelli et al., 2017; Ferreira et al., 2018). This is in line with a previous work indicating that the working experiences of mothers have a greater effect on family functioning relative to those of fathers. Such asymmetric gender patterns indicate that how mothers reconcile their work and family roles in the context of WFC have a greater impact on youth academic adjustment relative to similar reconciliation for fathers (Fuligni and Zhang, 2017; Aazami et al., 2018; Fan and Chen, 2020). This may also be linked to the traditional gender roles which predominate in China, wherein women are expected to be responsible for maintaining an appropriate work-family balance such that they effectively prioritize parenting while remaining strong participants in the workforce. Even in dual-earner families, mothers still tend to serve as the primary caregivers for children (Fontaine and Andrade, 2007). As such, maternal perceptions regarding difficulties in achieving work-family balance are linked to maladjustment in their children. Based on these findings, it is important to offer further impetus for workplaces and public policy to provide optimal employment conditions to mothers of children, by increasing parental employees satisfaction and reducing the experience of WFC (e.g., understanding of the needs of parents or providing greater flexibility in terms of their working hours, location, and work arrangements especially for working mothers).

We did not observe any evidence suggesting that paternal WFC can predict academic adjustment in adolescents. This may reflect the lesser role traditionally played by men when caring for children together with the greater ideological compatibility of employment and fatherhood (Bianchi and Milkie, 2010). Traditional Chinese culture values “breadwinning men and homemaking women,” which can make it challenging for fathers to take responsibility for their families. However, Schnittker (2007) determined that paternal WFC can reduce benefits to children under age 10, suggesting that gender differences may be accrued as men and women age and pass through different child-rearing stages (Leupp, 2017). Thus, future studies should seek to expand the current understanding of the association between work-family spillover and adolescent development, with a particular focus on fathers. We believed that should prove an interesting topic of investigation for a later study.

### Work-Family Enrichment and Academic Adjustment

Unlike under WFC, only a single indirect link between WFE and academic adjustment was detected for mothers. Specifically, no partner effect was evident under this model, with only actor effects of maternal educational expectations being observed in our study. In contrast to previous studies, these findings suggest that work and family obligations are not always at odds with one another such that for mothers who experience more WFE, spending more time with their children, and associated caregiving may be associated with better adolescent adaptability (Conger and Donnellan, 2007; Lee et al., 2017). Workforce participation can protect against common physical and mental health problems in mothers while increasing their opportunities for social support, income, skill-building, and identity, thereby contributing to feelings of efficacy and satisfaction (Grzywacz and Bass, 2003; Fontaine and Andrade, 2007; Bianchi and Milkie, 2010). These feelings, in turn, have the potential to strengthen maternal interactions with their children, yielding better adaptive outcomes. We believe that further studies should aim to dig more positive aspects of parent work-family spillover rather than just negative ones.

We did not find that positive maternal experiences impacted paternal expectations to the same extent as maternal conflict. This may be because husbands are less empathic to positive emotions and are thus more likely to experience negative affect from their spouse (Ong et al., 2010). For fathers, while fatherhood is important for adolescent development, unfortunately, at present, there is little such evidence for our studies.

### The Role of Educational Expectation

One mechanism whereby expectation impacts adolescent outcomes is *via* the mediating role of parental attitudes toward education and their engagement in learning activities and these impacts have long been used to explain individual actions and task performance (Lent et al., 2009; Crockett and Beal, 2012).

Our results suggest that parental educational expectations and perceptions thereof jointly mediate the relationship between parental work-family spillover and academic adjustment. Recent evidence further suggests that these expectations may explain to a large extent the association between the parental work-family interface and academic adjustment among adolescents. Our findings are consistent with such evidence (Yamamoto and Holloway, 2010; Vasquez et al., 2016). While parental educational expectations are external factors that are unable to directly impact academic adjustment in adolescents, parents nonetheless serve as the primary socializing agents for their children, transmitting their expectations and values in a manner that ultimately influences adolescent academic achievement (Yamamoto and Holloway, 2010; Vasquez et al., 2016).

Our results were in further support of a significant negative correlation between perceived educational expectations and academic adjustment among adolescents, consistent with the findings of Attanasio and Kaufmann (2014), who found higher levels of parental educational expectations to be closely linked to lower levels of adjustment (Attanasio and Kaufmann, 2014). In other words, perceived educational expectations have the potential to shape other outcomes *via* engendering academic stress (Haven et al., 2019; Poots and Cassidy, 2020). Self-cognition theoretically motivates behaviors while also being adjusted based upon feedback from those behaviors (Eccles and Wigfield, 2002; Beal and Crockett, 2010). Prior studies thus suggest that for adolescents, perceived parental educational expectations serve as an important reflective evaluation of adolescents. Indeed, adolescents reported more pressure and academic maladjustment when they perceived themselves as failing to meet the expectations of their parents (Bouchey and Harter, 2005; Agliata and Renk, 2008). The overestimation of parental expectations may indicate unsuccessful family socialization or maladaptive parent-child interactions, and such unfavorable family environments may compromise adolescent adjustment, highlighting important avenues for future intervention.

## Conclusion and Implications

While there have been many studies confirming the impact of parental work-family spillover on adolescent developmental outcomes, the effects of WFC and WFE on academic adjustment in adolescents are not well-understood in the Chinese cultural setting. This study is the first such empirical exploration of how such parental work-family spillover can positively and negatively influence such academic adjustment, and while these results are preliminary, they are nonetheless noteworthy. While fully clarifying the direct relationship between parental work-family spillover and child development remains challenging, our results suggest that only maternal expectations and perceived educational expectations mediate the relationship between these two variables. Specifically, our results suggest the potential for a crossover pattern within couples such that maternal WFC may adversely impact paternal expectations. As regards future research, more studies are needed to consider both work-family experiences of mothers and fathers and potential contributions to health inequalities.

These results indicate the potential for maternal work-family spillover to have both positive and negative impacts on the mental health of their children as well as their spouse (Sabbath et al., 2015; Romund et al., 2016; Vieira et al., 2016). As rates of maternal participation in the labor market continue to risk, these results indicate that a large proportion of children have the potential to be adversely or positively affected by workplace policies that influence such maternal work-family spillover. As such, policies must focus on facilitating a family-friendly work environment for working mothers.

Despite its novel contribution to the literature, our work has the following limitations. First, the data in this study were cross-sectional, precluding the potential to draw any causal inferences regarding the findings in this study. Thus, additional studies will be necessary to more fully understand the nature of the relationship between the level of coherence and the importance that adolescents attribute to work and family roles in daily life. Second, all of the measurements in this study were self-reported and are thus potentially subject to bias as a consequence of perceived social desirability. Finally, samples in this study were relatively homogenous given that this analysis focused only on adolescents from five full schools in Jiangxi, and additional work will thus be necessary to determine whether these results can be generalized to more diverse populations or in different cultures and regions. Our study also should be emphasized that the current sample included families from a middle-class background, and generalizability to other populations may be limited. In future studies, it may be useful to explore the impact of the parental work-family condition in rural samples and other ethnic groups, in which the linkages between work-family adjustments of parents and academic adjustment of adolescents may vary. Overall, our data suggest that family dynamics should be considered when evaluating adolescents and their families. Additionally, further studies will need to more fully elucidate the bidirectional relationships between adolescent adjustment and parental experiences.

## Data Availability Statement

The raw data supporting the conclusions of this article will be made available by the authors, without undue reservation.

## Ethics Statement

The studies involving human participants were reviewed and approved by the University Research Ethics Committee, Shaanxi Normal University, China. Written informed consent to participate in the study was provided by the participants’ legal guardian/next of kin.

## Author Contributions

XLW and LZ were responsible for the formulation of the research program. In addition, XLW was responsible for data collection, processing, and manuscript writing. XJW was responsible for the manuscript review. MZ was responsible for the revision of this manuscript. LZ was responsible for funding acquisition. After consultations and all the authors agreed with the rearrangement of the names. In the final version of the article, LZ is tagged as corresponding author.

## Conflict of Interest

The authors declare that the research was conducted in the absence of any commercial or financial relationships that could be construed as a potential conflict of interest.

## Publisher’s Note

All claims expressed in this article are solely those of the authors and do not necessarily represent those of their affiliated organizations, or those of the publisher, the editors and the reviewers. Any product that may be evaluated in this article, or claim that may be made by its manufacturer, is not guaranteed or endorsed by the publisher.
